# The expanding role of the erector spinae plane block: from concept to clinical integration

**DOI:** 10.1016/j.bjane.2025.844644

**Published:** 2025-05-17

**Authors:** João Pedro Fernandes Gonçalves, Anita Perpétua Carvalho Rocha de Castro, Luiz Gustavo Albuquerque, Thiago Ramos Grigio, Durval Campos Kraychete

**Affiliations:** a Universidade Federal da Bahia, Salvador, BA, Brazil; b Hospital Santa Izabel, Salvador, BA, Brazil; c Universidade de São Paulo, Departamento de Anestesiologia, São Paulo, SP, Brazil

## Why ESPB matters

The Erector Spinae Plane Block (ESPB) was first described in 2016 by Forero et al. as an innovative technique for treating chronic thoracic neuropathic pain.^1^ Since then, it has gained prominence as a regional anesthesia technique, competing with other peripheral blocks, such as the paravertebral block.^2^ As of February 2025, more than 1,700 studies have been published on ESPB, making it one of the most studied blocks today.^3^

ESPB offers an excellent safety profile compared to other regional blocks, as it is a superficial technique easily visualized under ultrasound. Moreover, the vertebral transverse process provides an anatomical barrier that minimizes risks commonly observed in other regional blocks, such as the pneumothorax associated with the paravertebral.^4^ Also, it has a shallow endpoint providing a superficial and compressible location, preventing significant bleeding.^2^

Its first successful clinical application was in the treatment of thoracic pain in patients with metastatic cancer and rib fractures. Still, since then, its indications have expanded to include postoperative analgesia and the management of acute and chronic pain.^5^ Its application along the entire spinal column allows for analgesia in cervical, thoracic, abdominal, and limb surgeries^3^ (Table 1). Additionally, ESPB has been increasingly incorporated into multimodal analgesia protocols, particularly in thoracic, abdominal, and orthopedic surgeries, where its opioid-sparing effect and favorable safety profile contribute to Enhanced Recovery After Surgery (ERAS) pathways.^6^ Moreover, ESPB can be delivered via single-shot injection or continuous infusion through a catheter, offering flexibility to meet procedural and patient-specific needs.^6^ This editorial coincides with the publication of four new meta-analyses and a novel randomized controlled trial on ESPB in this Brazilian Journal of Anesthesiology issue, further emphasizing the block’s clinical importance and evolution.^7^, ^8^, ^9^, ^10^, ^11^

**Table 1:** Applications of the Erector Spinae Plane Block (ESPB) by spinal level and volume range. (Adapted from Pawa et al., 2023).^6^

Level	Volume (mL)	Clinical Indications
Cervical (C7‒T1)	10‒20	Neck pain, thyroid surgery
Upper Thoracic (T2‒T3)	15‒25	Breast surgery, upper thoracic neuropathic pain
Mid-Thoracic (T4‒T6)	20‒30	Cardiac surgery, rib fractures
Lower Thoracic (T7‒T9)	20‒30	Upper abdominal procedures
Upper Lumbar (L1‒S1)	20‒25	Lower abdominal surgery, hip and lower limb procedures

## What We Know (and Don’t Know)

In the pediatric population, one systematic review evaluated the use of ESPB compared to Caudal Epidural Block (CEB) for abdominal surgeries in children.^7^ The findings demonstrated non-inferiority of ESPB in terms of analgesic efficacy, positioning it as a valuable alternative in pediatric regional anesthesia. ESPB emerged as a safe and effective technique, particularly relevant when minimizing procedural risk is essential. Unlike CEB, which involves neuraxial access, the ESPB target site is anatomically distant from the spinal cord, significantly reducing the risk of complications such as dural puncture, epidural hematoma, and systemic toxicity. Moreover, CEB is associated with adverse effects, including hypotension, arrhythmias, urinary retention, and motor blockade, especially when used with general anesthesia or when inadvertent systemic absorption occurs. Although ESPB requires greater technical expertise, especially regarding ultrasound-guided execution, its safety profile and versatility make it an appealing option for thoracic, abdominal, inguinal, hip, and femur surgeries in children.^12^ This makes ESPB a pragmatic choice in settings where CEB is contraindicated or less desirable due to its side-effect profile or invasiveness.

In pediatric cardiac surgeries**,** a meta-analysis included three randomized trials and two observational studies.^10^ The findings demonstrated a significant reduction in opioid use within the first 48 hours postoperatively in the ESPB group, suggesting that the technique may serve as an effective component of multimodal analgesia aligned with Enhanced Recovery Strategies (ERAS). This effect was achieved with a low incidence of adverse events, supporting the safety profile of ESPB even in high-risk pediatric cardiac populations. In addition to reducing opioid consumption, ESPB may also contribute to minimizing the neuroendocrine, metabolic, and immunological stress responses triggered by surgical trauma. These responses have been recognized as important risk factors for adverse postoperative outcomes, including delayed recovery and increased susceptibility to complications. ESPB could play a complementary role in optimizing perioperative management under Enhanced Recovery After Surgery (ERAS) protocols in pediatric cardiac surgery by attenuating the systemic stress response.^10^

In herpes zoster-related pain, a meta-analysis explored the role of ESPB in both acute neuritis and established Postherpetic Neuralgia (PHN).^9^ Although the included studies were heterogeneous in design and methodology, the findings consistently favored ESPB in reducing pain scores and overall analgesic consumption. When performed during the acute phase of herpes zoster, ESPB was associated with a lower incidence of progression to PHN, potentially preventing the development of chronic pain resulting from neuroplastic changes in the peripheral and central nervous system.^13^ In patients with established PHN, ESPB led to reductions in the use of gabapentinoids, paracetamol, and tramadol, highlighting its role in symptom control and minimizing polypharmacy. The mechanism likely involves somatic and sympathetic blockade, contributing to peripheral and central sensitization modulation. Additionally, the technical simplicity and favorable safety profile of ESPB make it particularly suitable for elderly and immunocompromised patients, who constitute a significant proportion of individuals affected by herpes zoster.

In a meta-analysis of cesarean sections, ESPB was compared to the Transversus Abdominis Plane (TAP) block for postoperative analgesia. ESPB was associated with prolonged analgesic duration, greater patient satisfaction, and lower opioid consumption in the immediate postoperative period. These findings are clinically relevant, especially in obstetric settings where minimizing opioid exposure supports enhanced maternal recovery and neonatal safety. The posterior approach of ESPB may offer more extensive and reliable analgesia by affecting both somatic and visceral components of the surgical pain, in contrast to the TAP block, which primarily targets the anterior abdominal wall. This broader dermatomal spread may contribute to its superior performance in certain studies. Significantly, ESPB was associated with improved analgesic outcomes without increasing adverse events such as nausea, vomiting, or prolonged hospital stay, supporting its safety in the obstetric population.

In breast surgery, a randomized controlled trial compared the Erector Spinae Plane Block (ESPB) to the Pectoserratus Plane Block (PSPB) in patients undergoing mastectomy.^8^ PSPB was associated with lower postoperative consumption of tramadol and dipyrone, as well as reduced risk of chronic pain at three months. At six months, however, the two groups had similar pain outcomes. Although ESPB did not demonstrate superiority in this study, it remains a viable alternative in clinical scenarios where PSPB is not feasible, particularly in patients with altered anatomy, coagulopathy, or technical limitations for anterior plane blocks. ESPB offers practical advantages, such as performing the block in the lateral position, broader dermatomal spread, and a safer posterior approach. Notably, the similarity in pain outcomes between ESPB and PSPB at six months suggests that ESPB may still offer durable analgesic benefits in the long-term postoperative period. These attributes make it a valuable option within a multimodal analgesic strategy, even in procedures as challenging as mastectomy.

## Challenges in clinical implementation

Despite its promising clinical profile and growing evidence base, several practical barriers limit the widespread adoption of ESPB in routine anesthetic practice. One of the primary challenges lies in the need for proper ultrasound-guided training. The success of the block is highly dependent on operator skill and familiarity with sonoanatomy, and inadequate training can increase the risk of failure or suboptimal results.^14^ Additionally, there is significant institutional and cultural variability in accepting newer regional techniques. Many centers still favor more traditional blocks, such as paravertebral or epidural anesthesia, which may limit the integration of ESPB into perioperative pathways, especially in institutions with limited access to ultrasound machines or personnel proficient in their use.

Another relevant issue is the lack of standardization in clinical protocols. Variations in block technique (single-shot vs. catheter), puncture levels, and anesthetic volume and concentration have been documented across studies, contributing to heterogeneity and making it challenging to replicate outcomes reliably.^10^ Furthermore, in the context of herpes zoster-related pain, differences in block timing, dermatomal level, and the use of adjuvants have complicated the interpretation of ESPB’s isolated effects.^9^ Similarly, in cesarean deliveries, although ESPB showed promise over TAP block regarding analgesic duration and opioid-sparing effects, methodological differences and small sample sizes necessitate cautious interpretation of the results.^11^

More broadly, the inconsistency of injectable spread and the diversity of anesthetic regimens across studies introduce uncertainty regarding the reproducibility of ESPB’s clinical benefits. Short follow-up periods and the underreporting of functional and patient-centered outcomes hinder the establishment of robust evidence. These methodological challenges, combined with logistical and anatomical limitations in some surgical settings, emphasize the urgent need for high-quality, standardized, and adequately powered trials to better define the role of ESPB in modern regional anesthesia.

Moreover, ESPB presents anatomical and technical limitations for anterior thoracic procedures, such as sternotomies and thoracotomies. The posterior approach may not consistently provide sufficient coverage of the anterior chest wall, raising concerns about its adequacy in these settings.^2^ Furthermore, despite being considered a relatively safe technique, the effectiveness of ESPB heavily depends on operator expertise in ultrasound guidance and anatomical recognition, which introduces a non-negligible learning curve that may impact its reproducibility across different clinical environments.^14^ Consequently, some authors advocate for alternative regional techniques, including the pectoserratus plane block and parasternal block, to achieve more reliable anterior thoracic analgesia. In this context, a comparative overview of ESPB and other commonly used regional techniques is presented in Table 2, highlighting practical differences that may influence clinical adoption.

**Table 2:** Comparative Overview of Regional Blocks.^6^^,^^15^

Block	Difficulty of Execution	Analgesic Coverage	Risk of Complications	Typical Clinical Applications
Erector Spinae Plane Block (ESPB)	Easy	Multidermatomal, posterior and lateral	Low	Thoracic, abdominal, orthopedic, chronic and oncologic pain procedures
Thoracic Paravertebral Block (TPVB)	Moderate	Segmental, unilateral	Moderate (pneumothorax, hypotension)	Breast surgery, thoracotomy, rib fractures
Transversus Abdominis Plane Block (TAP)	Easy	Abdominal wall only	Low	Lower abdominal procedures, cesarean section, hernia repair
Pectoral Nerve Blocks (PECS I/II)	Easy	Anterior thoracic wall	Low	Mastectomy, chest wall surgery
Rectus Sheath Block	Easy	Midline anterior abdomen	Low	Umbilical hernia, laparoscopic access analgesia
Caudal Epidural Block (CEB)	Easy	Subumbilical dermatomes	Moderate (hypotension, arrhythmias)	Urological, gynecological, lower limb procedures

Finally, the economic and logistical aspects must be considered. While the ESPB has the advantage of being relatively safe and straightforward, its implementation requires investment in equipment, training programs, and workflow adjustments, barriers that may disproportionately affect low-resource settings. Addressing these challenges will require coordinated efforts in education, research, and institutional support to ensure that ESPB can be safely and effectively integrated into multimodal analgesia strategies.

## Where we go from here

Looking ahead, several strategies may help strengthen the role of ESPB in clinical practice. First, future research should prioritize high-quality, multicenter randomized controlled trials to confirm the block’s efficacy in various surgical scenarios, including those involving chronic pain, oncologic pain, and anterior thoracic approaches. Additionally, more extended follow-up periods and the inclusion of functional, patient-centered outcomes are essential to fully capture the clinical impact of ESPB.

Given its versatility, being performable at any spinal level and applicable in thoracic, abdominal, orthopedic, and chronic pain procedures, ESPB stands among the seven 'plan A' blocks advocated for routine use in regional anesthesia.^6^ Its ability to serve as a single-shot and catheter-based continuous infusion makes it adaptable to a wide range of perioperative needs.

Therefore, inequity in access must be addressed. ESPB requires ultrasound guidance, so its adoption may be limited in low-resource settings. Policy and institutional initiatives should aim to reduce these disparities through expanded training programs and infrastructure support. Finally, clinicians must continue to evaluate each case individually. While ESPB offers an appealing combination of efficacy and safety, it is not universally applicable. Patient characteristics, surgical demands, and clinician expertise should always guide the selection of regional techniques. With thoughtful implementation and continued research, ESPB may establish itself as a cornerstone of regional anesthesia, bridging simplicity and safety across a wide range of surgical and pain management scenarios.

## Authors’ contributions

João Pedro Fernandes Gonçalves: Conceptualization, writing-original draft, review & editing.

Anita Perpétua Carvalho Rocha de Castro: Writing-review & editing, supervision.

Luiz Gustavo Albuquerque: Writing-review & editing.

Thiago Ramos Grigio: Writing-review & editing.

Durval Campos Kraychete: Supervision, critical review of the manuscript.

## Institutional review board approval

Not applicable.

## Study registration

Not applicable.

## Conflicts of interest

The authors declare no conflicts of interest.
